# Editorial: Epilepsy and endocrine function

**DOI:** 10.3389/fendo.2023.1288784

**Published:** 2023-09-28

**Authors:** Barbara Miziak, Stanisław J. Czuczwar

**Affiliations:** Department of Pathophysiology, Medical University of Lublin, Lublin, Poland

**Keywords:** epilepsy, hormone, ketogenic diet, osteoporosis, seizures

The relationship between epilepsy and endocrine functions seems evident. Prior studies suggest that seizure activity affects the sex steroid hormone axis, which in turn results in female and male sexual dysfunction, namely lower fertility (1). Historically, progesterone was used effectively as an adjuvant treatment for catamenial epilepsy (2). Several antiseizure medications (ASMs) have been documented to influence the release of sex hormones, their metabolism, and their interactions with proteins in the blood (1, 3). Some ASMs (for example, carbamazepine, oxcarbazepine, phenobarbital, phenytoin, topiramate) have been shown to negatively impact bone health by reducing intestinal calcium absorption, increasing calcium mobilization from the skeleton and decreasing bone mineralization (4). Thus, both a history of epilepsy and prescribed ASMs modulate the association between seizures and hormonal pathophysiology. Interestingly, among neuroactive steroids, there are two groups of compounds that either positively (e.g. allopregnanalone) or negatively (e.g. pregnanolone sulfate) modulate GABA_A_ receptor-mediated events. Positive modulators exhibit anticonvulsant effects as evaluated in animal models of seizures. They possess a potential for the development of efficient ASMs (5). Among non-pharmacological treatments of epilepsy, ketogenic diet may play a considerable role, especially in pediatric patients. Indeed, its effect on the hypothalamic-pituitary-adrenal axis and the renin-angiotensin-aldosterone system was found (6, 7).

Brief summaries of the publications within this Research Topic follow:

Observational studies clearly indicate that patients with epilepsy who are prescribed ASMs are at risk of developing secondary osteoporosis with associated reduced mineral bone density (4). It is possible that the osteoporotic potential of a number of ASMs may be further potentiated by other drugs (e.g. glucocorticoids or proton pump inhibitors) taken by patients with epilepsy for concurrent comorbidities (8). Tang et al. have undertaken studies to elucidate a relationship between neurologic diseases, including epilepsy, and osteoporosis. Their analysis of over 53,400 patients with epilepsy, which utilized Mendelian randomization and inverse variance weighting-random effects, did not show any direct evidence to link epilepsy with an increased risk of osteoporosis. The authors note a discrepancy between their conclusions and results of the observational studies discussed previously, indicating that observational results might have been confounded by other risk factors.

The next study has been devoted to epileptic seizures associated with non-ketotic hyperglycemia as seen among diabetic patients (Baltyde et al.). The authors describe a detailed series of 18 patients in regard to their clinical, biological, radiological data, as well as length of hospitalization. This retrospective study suggests that patients suffering from non-ketotic hyperglycemia do not require ASMs and their seizures would stop within 24 hours following rehydration and insulin therapy.

Bibliometric analysis, performed by Wang et al., aimed at revealing the status of ketogenic diet during 2000-2021 observation period. The authors conducted a retrospective analysis of 2808 publications which were published and cited 89,899 times within this 11-year time frame. This analysis shows that the number of publications on the topic of ketogenic diet has been sharply increasing over time, from less than 50 in 2000 to over 300 in 2021. A similar trend is noted in the number of citations, from just 200 in 2000 to over 16,000 in 2021. This analysis also provides insights into the most common countries, organizations, and authors on the topic, as well as the journals in which this work is most commonly published. The authors also note that ketogenic diet is becoming a more favored therapeutic option for disease processes beyond childhood epilepsy, including adult epilepsy and other neurological diseases such as Parkinson’s disease, Alzheimer’s disease, and other neurodegenerative diseases.

The last publication in this Research Topic is a case report of infantile spasms in a small-for-gestational-age infant with low plasma glucose concentration (Chandran et al.). Seizures were resistant after correction of glucose insufficiency. MRI showed cytotoxic cerebral edema. Diazoxide was started and discontinued at 4 months of life. At the age of 5 months, seizure activity was observed again and the treatment with high dose of oral prednisolone was initiated. The authors discuss this case and compare to the available cohort studies of infants with hypoglycemic brain injury.

This Research Topic encompasses original, review, and case-report studies which broaden the knowledge of the existing association between seizures and endocrine activity. The results may be helpful for conducting proper therapeutic strategies and providing deeper insight into ketogenic diet and risk of osteoporosis in patients with epilepsy.

## Author contributions

BM: Conceptualization, Writing – original draft, Writing – review & editing. SC: Conceptualization, Writing – original draft, Writing – review & editing.
